# Age‐Related Anatomical Changes in Carotid Artery Stenosis and Its Impact on Postoperative Complications in Stenting and Endarterectomy

**DOI:** 10.1111/cns.70527

**Published:** 2025-07-23

**Authors:** Xiao Zhang, Jia Zhou, Renjie Yang, Jiaqi Jin, Yong Zeng, Shuaiwei Guo, Jiayao Li, Yixin Sun, Zixuan Xing, Shengyan Cui, Xinyu Yang, Xiangyu Li, Wenjing Li, Xiaoli Min, Liqun Jiao, Tao Wang

**Affiliations:** ^1^ Department of Neurosurgery, Xuanwu Hospital Capital Medical University Beijing China; ^2^ China International Neuroscience Institute (China‐INI) Beijing China; ^3^ State Key Laboratory of Complex Severe and Rare Diseases, Peking Union Medical College Hospital Chinese Academy of Medical Sciences & Peking Union Medical College, National Clinical Research Center for Dermatologic and Immunologic Diseases Beijing China; ^4^ Department of Psychiatry The Second Affiliated Hospital of Kunming Medical University Kunming China; ^5^ First Hospital Peking University Beijing China; ^6^ Health Science Center Peking University Beijing China; ^7^ Health Science Center Xi'an Jiaotong University Xi'an Shanxi China; ^8^ Laboratory of Computational Biology and Machine Intelligence, National Laboratory of Pattern Recognition Institute of Automation, Chinese Academy of Sciences Beijing China; ^9^ School of Artificial Intelligence University of Chinese Academy of Sciences Beijing China; ^10^ Department of Cerebrovascular Diseases The Second Affiliated Hospital of Kunming Medical University Kunming China; ^11^ Department of Interventional Neuroradiology Xuanwu Hospital, Capital Medical University Beijing China

**Keywords:** age, anatomic profiles, carotid artery stenosis, endarterectomy, postoperative complications, stent

## Abstract

**Aims:**

Carotid artery stenosis increases the risk of ischemic stroke, with carotid endarterectomy (CEA) and carotid artery stenting (CAS) as primary interventions. Age‐related vascular changes may contribute to complications. This study aimed to evaluate the impact of age‐related vascular changes on postoperative complications and procedural outcomes.

**Methods:**

A retrospective cohort of 470 patients who underwent CAS or CEA from January 2020 to November 2021 was analyzed. Demographics, anatomical characteristics, and postoperative complications were assessed. Correlation, regression analyses, and machine learning models were applied to identify predictors of adverse outcomes.

**Results:**

Postoperative complications occurred in 64.9% of CAS and 75.2% of CEA patients. Older age correlated with larger CCA diameter, shorter clavicle‐to‐bifurcation distance, and increased tortuosity of both CCA and ICA. Several age‐related anatomical changes were significantly linked to higher complication rates in both procedures. In CAS, key predictors included symptomatic stenosis, aortic arch variation, CCA ostial lesions, and CCA diameter (*p* < 0.05). A logistic regression model predicted CAS complications effectively (AUC = 0.82).

**Conclusion:**

This study highlights significant age‐related changes in carotid artery anatomy and their impact on postoperative complications. These findings underscore the importance of considering age‐related vascular remodeling to enhance patient selection and optimize surgical outcomes.

## Graphical abstract

Using a clinical cohort of 429 patients, this study identified age‐related carotid anatomical changes—such as increased tortuosity, arterial stiffness, and CCA diameter variation—that were significantly associated with CAS/CEA complications. A logistic regression model accurately predicted CAS complications (AUC = 0.82), outperforming multiple machine learning methods. Findings may aid in optimizing patient selection and surgical planning in the elderly.

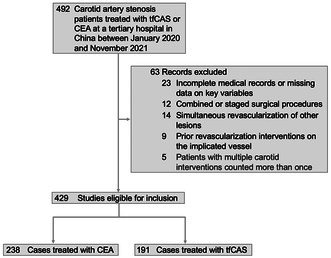

**Abbreviations**
AIC: Akaike information criterion
AUC: area under the ROC curve
BCA: brachiocephalic artery
BMI: body mass index
CAS: carotid artery stenting
CCA: common carotid artery
CEA: carotid endarterectomy
GBM: gradient boosting machines
ICA: internal carotid arteries
NASCET: North American Symptomatic Carotid Endarterectomy Trial
OR: odds ratios
RF: random forests
SVM: support vector machines
TIA: transient ischemic attack
VIF: variance inflation factor

## 1 Introduction

Carotid artery stenosis is a significant risk factor for ischemic stroke, with a 5‐year cumulative all‐cause mortality rate of 23.6% [1]. The internal carotid arteries (ICAs), branching from the common carotid arteries (CCAs) at the carotid sinus, provide crucial blood supply to the central nervous system. Surgical interventions for carotid artery stenosis include carotid endarterectomy (CEA) and carotid artery stenting (CAS), each with distinct procedural risks and benefits [2, 3, 4]. While CAS is less invasive than CEA, its adoption has been limited, partially due to a higher incidence of perioperative stroke, myocardial infarction, and mortality risks; however, long‐term ipsilateral stroke rates are comparable [5, 6, 7].

Age is not only a risk factor for the development of carotid artery stenosis [8, 9] but also a predictor of post‐procedural complications, posing challenges in treatment selection. A large meta‐analysis involving over 500,000 patients demonstrated that advanced age significantly increases the risk of stroke in patients undergoing CAS, whereas CEA showed comparable neurologic outcomes across age groups but was associated with increased mortality in older patients [10]. The ACST‐1 study reported that among patients over 75 years, half of those undergoing CEA did not survive beyond 5 years [11]. In CAS, advanced age has been associated with an increased risk of post‐procedural hyperperfusion syndrome and new ischemic brain lesions [12, 13]. Additionally, older age has been identified as an independent risk factor for restenosis following both CEA and CAS [14]. The SAPPHIRE trial, which randomized 334 high‐risk patients to CEA or CAS, included age > 80 years as a criterion for high surgical risk. At this risk level, most patients did not derive significant benefit from either procedure, supporting medical management as the preferred approach [15].

Age remains a significant limitation to the broader clinical adoption of CAS, despite its less invasive nature. An individual patient meta‐analysis by the Carotid Stenting Trialists Collaboration demonstrated a strong association between increasing age and a higher 30‐day risk of stroke or death following CAS, whereas this trend was not observed for CEA. Among patients over 70 years, CAS was associated with higher stroke and mortality rates compared to CEA, while outcomes were similar between the two procedures in patients younger than 70 [16]. The 2023 ESVS guidelines recommend CEA over CAS for symptomatic patients (i.e., those with a history of TIA or ischemic stroke within the past 6 months) with 50%–99% carotid stenosis if they are aged ≥ 70 years. For patients younger than 70, CAS may be considered as an alternative to CEA. However, performing CAS in the acute phase—particularly within 14 days—is associated with an elevated stroke risk, whereas CEA has demonstrated greater safety during this period [17]. Other studies have explored specific perioperative risk factors, such as postoperative hyperglycemia, identifying it as an independent predictor of symptomatic intracranial hemorrhage following endovascular therapy [18]. In addition, neuroprotective strategies such as remote ischemic conditioning have shown promise in improving cerebrovascular outcomes and reducing complications in stroke patients, including those undergoing interventional treatments [19]. While these studies have contributed important insights into perioperative risk assessment and intervention strategies, most existing models focus primarily on clinical or biochemical parameters. In contrast, our study integrates age‐related anatomical changes in carotid vasculature, such as CCA diameter, bifurcation distance, and tortuosity, as novel predictive variables for postoperative complications in both CAS and CEA. To our knowledge, this is among the first studies to comprehensively quantify vascular remodeling associated with aging and incorporate these anatomical features into a predictive framework. This approach provides a more anatomically grounded and individualized risk stratification strategy, with the potential to inform both preoperative planning and patient selection.

The mechanisms by which age influences patient survival through intermediary variables remain an important area for further research. While age has been shown to increase the risk of postoperative complications, this effect appears to be more pronounced in CAS. Some studies suggest that this may be attributed to factors such as increased atherosclerotic burden, aortic arch calcification, vascular anatomical changes, and greater plaque vulnerability; these age‐related vascular alterations and disease progression may contribute to arterial stiffness or tortuosity, making CAS guidewire navigation more challenging and increasing the risk of embolization and perioperative stroke. However, these hypotheses lack evidence from clinical studies [20]. Additionally, research has demonstrated that anatomical changes, including increased vessel volume, bifurcation angle, and enlargement of the carotid artery and ICA, also progress with age, yet it did not link these age‐related vascular changes in postoperative complication rates [21, 22]. Overall, there is still a lack of studies exploring how these anatomical features mediate the association between age and postoperative complications.

This study aimed to investigate the relationship between age and carotid artery anatomical characteristics, focusing on changes in vessel diameter, bifurcation level, and tortuosity. Furthermore, we evaluate how these anatomical factors influence postoperative complications in patients undergoing CEA and CAS. By identifying age‐related anatomical predictors of complications, our findings may help optimize patient selection and treatment strategies to improve outcomes.

## 2 Methods

### 2.1 Study Population

**FIGURE 1:**
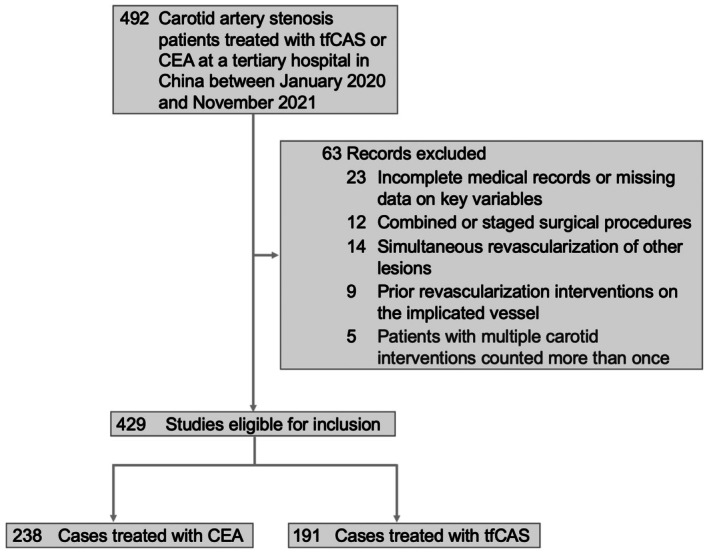
The flow chart of population collection. This figure illustrates the recruitment process, including the inclusion and exclusion criteria applied, the number of participants at each stage, and the final sample size utilized for analysis.

This retrospective cohort study adheres to the STROBE checklist and was conducted using data from the Department of Neurosurgery at a tertiary hospital, encompassing patients diagnosed with carotid artery stenosis who received either CEA or CAS between June 2020 and November 2021. Inclusion criteria required patients to have a diagnosis of carotid artery stenosis and to have undergone one of the aforementioned surgical interventions (Figure 1). Carotid artery stenosis was assessed using computed tomography angiography according to the North American Symptomatic Carotid Endarterectomy Trial (NASCET) criteria, which involves comparing the diameter of the most stenotic segment with that of the normal distal internal carotid artery. Stenosis was classified as mild (< 50%), moderate (50%–69%), severe (≥ 70%), or complete occlusion (100%) [23]. Symptomatic stenosis was defined by the occurrence of neurological symptoms within 6 months, including transient ischemic attack (TIA) or stroke, confirmed through clinical evaluation and imaging.

Patients were excluded if their medical records were incomplete, data on key variables were missing, or they underwent combined or staged surgical procedures. Patients with simultaneous revascularization of other lesions or prior revascularization interventions on the implicated vessel were also excluded. To ensure accurate analysis, patients who underwent multiple carotid interventions during the study period were not counted more than once.

The cohort was stratified based on the side of stenosis to account for anatomical differences between the left and right common carotid arteries. Patients were divided into two groups: the left (left CCA stenosis) and the right (right CCA stenosis). Each group underwent independent and comprehensive analyses. This stratification was crucial due to the significant anatomical differences between the two sides, which required separate analyses to ensure the reliability of the results. Data from each group were analyzed using consistent procedures to elucidate the impact of side‐specific carotid stenosis on anatomical parameters and postoperative complications.

Data were systematically extracted from electronic medical records. Baseline characteristics collected included age, gender, body mass index (BMI), smoking, and alcohol use history, and comorbidities. Postoperative complications were recorded within 30 days, focusing on stroke, myocardial infarction, and cranial nerve injury. Stroke was defined as new neurological deficits (NIHSS increase ≥ 4, persisting > 24 h). Asymptomatic infarcts were identified via MRI diffusion‐weighted imaging. Acute myocardial infarction was diagnosed based on chest symptoms, shock, or heart failure, ECG changes (Q waves, ST‐segment elevation, ST‐T changes), and elevated myocardial enzymes. These outcomes were assessed for recovery and prognosis impact.

### 2.2 Anatomic Characteristics and Age‐Related Changes

Prior studies identified age‐related anatomical changes, including increased vessel volume, CCA and ICA diameters, bifurcation angle, and carotid artery widening/rotation. Conversely, aging is linked to decreased wall shear stress, velocity, pressure gradient, and energy loss [21, 22]. We collected anatomical parameters—CCA diameter, proximal/distal ICA diameter, CCA depth above the clavicle, distance to carotid bifurcation, CCA bifurcation location, and CCA/ICA tortuosity index—for correlation analysis.

CT angiography evaluated aortic and carotid anatomy, analyzing surgical impact and complications. Aortic arch classification was based on the apex‐to‐BCA origin distance: Type I (≤BCA width), Type II (>BCA width, ≤ 2 × BCA width), and Type III (> 2 × BCA width), influencing procedural complexity [24, 25]. Variations in the aortic arch were also noted, such as the “bovine arch” found in approximately 7%–20% of individuals [25, 26].

The diameter of the CCA was measured 15–20 mm below the bifurcation and without significant plaques [27]. For assessing the proximal ICA diameter, the NASCET standards were applied [23, 28, 29]. Measurements of the distal ICA diameter were taken just before the petrous bend [29, 30]. Tortuosity indices for both the CCA and ICA were determined by summing the deviation angles from a straight line in the imaging projection [31]. CCA bifurcation was referenced to vertebral body level. CCA depth above the clavicle assessed arterial access difficulty, especially in deeper necks [32]. The distance between the clavicle and carotid bifurcation was assessed to consider the angled trajectory required for accessing the artery [32].

Aortic arch calcification was categorized into four grades: Grade 0 (no visible calcification), Grade 1 (small spots or thin areas), Grade 2 (one or more thickened areas), and Grade 3 (circular calcification around the aortic knob) [33, 34]. CCA ostial lesions were examined for stenosis within 3 mm of the origin of the artery [35]. Ostial disease differs from intravascular lesions, necessitating separate analyses due to treatment implications [36]. CCA puncture‐site lesions (calcification above the clavicle) were graded none/minimal versus moderate/severe [32]. Tandem lesions, which are defined as significant stenosis or occlusion in the cervical ICA combined with thromboembolic occlusion in the intracranial ICA or middle cerebral artery, were noted if any stenosis exceeded 50% [37].

### 2.3 Statistical Analysis

To maximize the information obtained from the data, age was analyzed as a continuous variable, along with height, weight, and BMI, while gender was treated as a binary categorical variable. Normality was assessed using the Shapiro–Wilk test, with a *p* value of less than 0.05 indicating a non‐normal distribution. Pearson's correlation was used to explore associations between continuous independent variables (e.g., age, height, weight, and BMI) and continuous anatomical characteristics (e.g., diameter of the CCA), assuming normality of the data. For anatomical parameters or independent variables that did not meet the assumption of normality, Spearman correlation was applied. Gender‐related differences in continuous anatomical variables (e.g., diameter of the CCA) were assessed using the independent *t*‐test for normally distributed data and the Wilcoxon rank‐sum test for non‐normally distributed data. We considered both indicators of disease severity (including symptomatic stenosis, variations in the aortic arch, CCA ostial lesions, aortic arch calcification level, and tandem lesions) and age‐related anatomical changes (including diameter of the CCA, proximal ICA diameter, distal ICA diameter, depth of the CCA above the clavicle, distance between the clavicle and carotid bifurcation, location of the CCA bifurcation, and tortuosity index for CCA and ICA) as dependent variables. Univariate logistic regression was conducted to evaluate their impact on postoperative outcomes. A *p* value of < 0.05 indicated statistical significance, and odds ratios (OR) greater than 2.0 or less than 0.8 were considered to have practical or clinical significance, justifying their inclusion in the multivariate model. Significant variables from univariate analysis were subjected to multicollinearity checks using the variance inflation factor (VIF), with a VIF > 10 suggesting multicollinearity. Stepwise multivariate regression was then performed, guided by the Akaike information criterion (AIC), to select the optimal model by excluding insignificant or redundant variables. Model performance was evaluated using the area under the ROC curve (AUC ≥ 0.8), with a calibration curve used to assess how well the predicted probabilities matched the observed outcomes. Decision curve analysis was employed to evaluate clinical utility, with a higher net benefit curve across thresholds, compared to the “ALL” and “NONE” models, indicating strong clinical applicability. In addition to logistic regression for prediction, various machine learning algorithms were employed, including the decision tree, support vector machines (SVM), random forests (RF), and gradient boosting machines (GBM), to compare the predictive performance of these methods.

## 3 Results

### 3.1 Baseline Characteristics

A total of 470 patients (comprising 895 cases of carotid artery stenosis) were included in this cohort (Table S1). Among them, 449 patients presented with right‐sided stenosis, while 446 patients had left‐sided stenosis. The mean age of the patients was 64.6 ± 8.1 years. The majority of the cohort was male (*n* = 406, 86.4%), with females accounting for 13.6% (*n* = 64). The average height was 167.8 ± 8.0 cm, and the average weight was 71.4 ± 11.5 kg, resulting in a mean BMI of 25.8 ± 15.0 kg/m [2].

Cardiovascular diseases (e.g., hypertension, coronary artery disease, peripheral vascular disease, history of atrial fibrillation, and history of heart failure) were highly prevalent in this cohort, affecting 75.5% of the patients (*n* = 355), while metabolic disorders were present in 64.7% (*n* = 304). The incidences of respiratory diseases (0.6%, *n* = 3), gastrointestinal conditions (1.1%, *n* = 5), renal or hepatic dysfunction (0.6%, *n* = 3), and hematologic disorders (0.2%, *n* = 1) were low.

In terms of smoking status, 42.8% of patients (*n* = 201) had never smoked, 31.5% (*n* = 148) were current smokers, and 25.7% (*n* = 121) were former smokers. Regarding alcohol consumption, 55.5% of patients (*n* = 261) had never consumed alcohol, 31.7% (*n* = 149) were current drinkers, and 12.8% (*n* = 60) were former drinkers.

Of the total cohort, 429 patients had detailed surgical records. Among them, 191 underwent CAS, while 238 underwent CEA. Postoperative complications occurred in 64.9% of CAS patients and 75.2% of CEA patients. The complication rate was significantly lower in the CAS group compared to the CEA group (*p*‐value = 0.027).

### 3.2 Age‐Related Anatomic Characteristics in the Carotid Artery

The aortic arch was classified into three types: Type I was observed in 43.2% of cases (*n* = 203), Type II in 27.8% (*n* = 131), and Type III in 28.9% (*n* = 136). Aortic arch variants were identified in 12.1% of patients (*n* = 57). The mean tortuosity index for the CCA was 116.6 ± 60.4, and for the ICA, it was 150.3 ± 70.2. The cohort was divided into left‐side and right‐side stenosis groups to assess anatomical characteristics and their correlations with age, sex, height, weight, and BMI. The results from both groups were mostly consistent, indicating the robustness of the data (Table 1).

**TABLE 1:** Correlation analysis between anatomical characteristics and demographic information.

Anatomic characteristics	Demographic characteristics								
	Age (year)		Gender	Height (cm)		Weight (kg)		BMI	
	*ρ*	*p*	*p*	*ρ*	*p*	*ρ*	*p*	*ρ*	*p*
Diameter of the CCA (right) (mm)	**0.094**	**0.047**	0.097	−0.038	0.43	**0.118**	**0.012**	**0.148**	**0.002**
Diameter of the CCA (left) (mm)	0.065	0.17	0.37	−0.046	0.34	**0.118**	**0.013**	**0.162**	**< 0.001**
Depth of the CCA above the clavicle (right) (cm)	−0.017	0.71	0.14	0.018	0.71	**0.243**	**< 0.001**	**0.272**	**< 0.001**
Depth of the CCA above the clavicle (left) (cm)	0.045	0.35	0.97	−0.034	0.47	**0.147**	**0.002**	**0.190**	**< 0.001**
Distance between the clavicle and carotid bifurcation (right) (cm)	**−0.095**	**0.045**	**< 0.001**	**0.184**	**< 0.001**	**0.096**	**0.041**	0.003	0.95
Distance between the clavicle and carotid bifurcation (left) (cm)	**−0.145**	**0.002**	**< 0.001**	**0.217**	**< 0.001**	**0.136**	**0.004**	0.022	0.64
Location of the CCA bifurcation (right)	−0.065	0.17	0.82	0.014	0.77	−0.053	0.26	0.020	0.68
Location of the CCA bifurcation (left)	−0.017	0.73	0.49	0.011	0.82	−0.076	0.11	−0.004	0.94
Proximal ICA diameter (right) (mm)	−0.036	0.45	0.082	0.077	0.10	0.064	0.18	0.033	0.49
Proximal ICA diameter (left) (mm)	0.008	0.86	0.12	**0.161**	**< 0.001**	**0.221**	**< 0.001**	**0.177**	**< 0.001**
Distal ICA diameter (right) (mm)	0.075	0.11	0.40	0.004	0.94	0.059	0.21	0.057	0.23
Distal ICA diameter (left) (mm)	−0.007	0.89	0.39	0.074	0.12	**0.148**	**0.002**	**0.115**	**0.016**
Tortuosity index for CCA (right)	**0.222**	**< 0.001**	**< 0.001**	**−0.228**	**< 0.001**	0.077	0.10	**0.263**	**< 0.001**
Tortuosity index for CCA (left)	**0.160**	**< 0.001**	**0.012**	**−0.155**	**0.001**	**0.134**	**0.005**	**0.282**	**< 0.001**
Tortuosity index for ICA (right)	**0.202**	**< 0.001**	0.17	**−0.163**	**< 0.001**	0.045	0.34	**0.159**	**< 0.001**
Tortuosity index for ICA (left)	**0.207**	**< 0.001**	0.058	**−0.102**	**0.032**	0.060	0.21	**0.153**	**0.001**

*Note:* The bold values in Table 1 indicate statistically significant results (*p* < 0.05).

Abbreviations: BMI, body mass index; CCA, common carotid artery; ICA, internal carotid artery.

**FIGURE 2:**
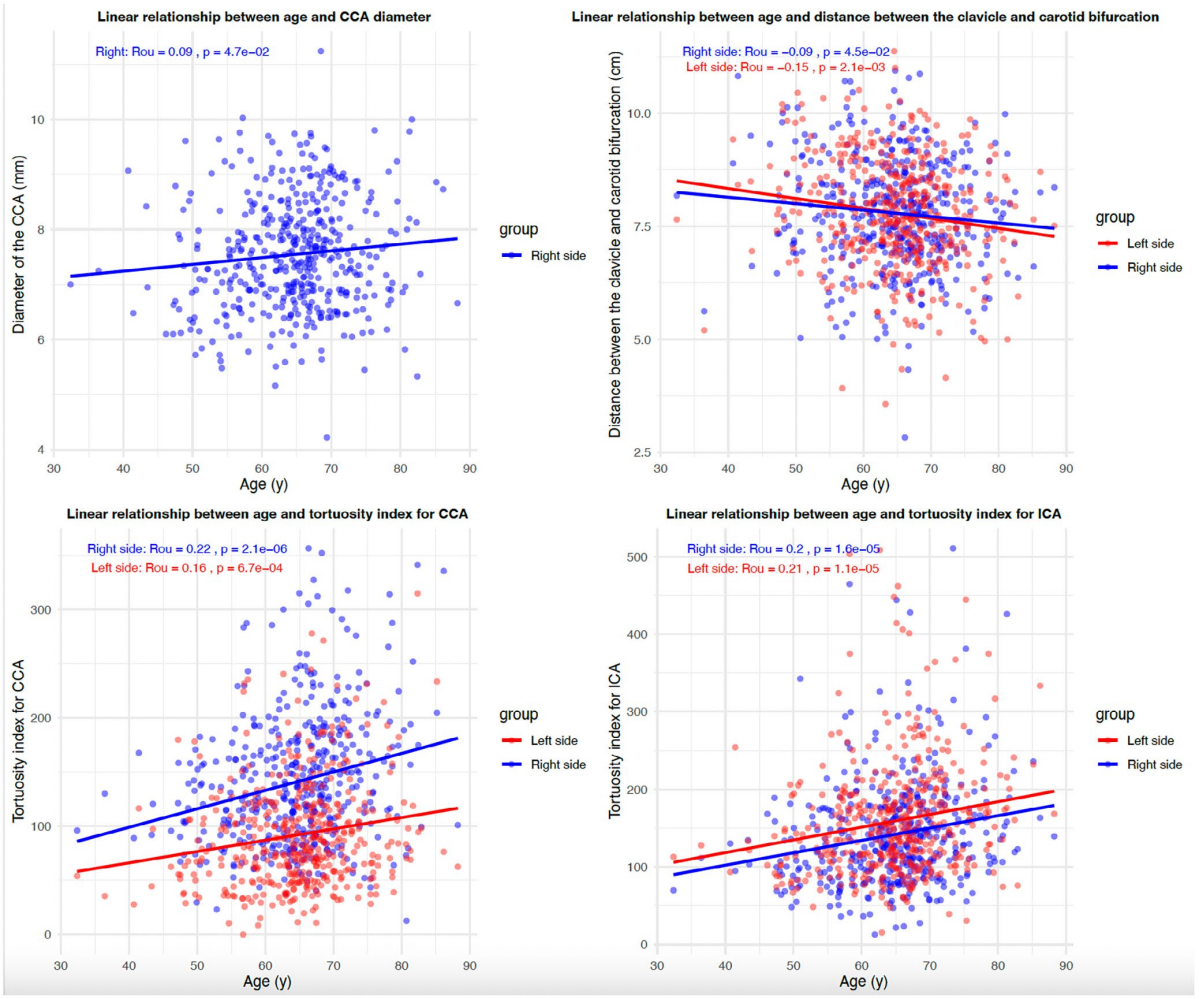
The correlation between several anatomic features and age with significance. This figure illustrates the significant associations between age and various anatomical characteristics, including the diameter of the common carotid artery (only at the right side), the distance between the clavicle and the carotid bifurcation, and the tortuosity indices for both the common carotid artery and internal carotid artery. Data are presented as scatter plots with regression lines. Statistical significance is indicated.

Several anatomic features, including the diameter of the CCA, the distance between the clavicle and carotid bifurcation, and the tortuosity indices for both the CCA and ICA, were significantly associated with age and exhibited age‐related changes (Figure 2). The diameter of the CCA increases with age, while the distance between the clavicle and carotid bifurcation decreases with age. Additionally, the tortuosity index for both the CCA and ICA rises as age increases. Notably, the diameter of the CCA was only significantly correlated with age in the right‐side stenosis group, whereas other features showed significant correlations with age on both sides. Height was significantly associated with the distance between the clavicle and carotid bifurcation, proximal ICA diameter, and the tortuosity indices for both the CCA and ICA. While the proximal ICA diameter was only significantly correlated with height in the left‐side stenosis group, the other features were significant for both sides. Weight significantly impacted several anatomic features, including the diameter of the CCA, the depth of the CCA above the clavicle, the distance between the clavicle and carotid bifurcation, and the proximal and distal ICA diameters on the left side. The tortuosity index for the CCA was significantly related to weight in the left‐side stenosis group. BMI was significantly associated with the diameter of the CCA, the depth of the CCA above the clavicle, the proximal and distal ICA diameters on the left side, and the tortuosity indices for both the CCA and ICA. Sex significantly affected the distance between the clavicle and carotid bifurcation and the tortuosity index for the CCA.

Further analysis revealed that the diameter of the CCA and the depth of the CCA above the clavicle increased with weight. The distance between the clavicle and carotid bifurcation was influenced by height, weight, and age, with larger measurements observed in taller and heavier patients and smaller measurements in older patients. The tortuosity indices for both the CCA and ICA were primarily influenced by age, height, and BMI, with increased tortuosity seen in patients with higher BMI, shorter stature, and advanced age. Notably, the proximal and distal ICA diameters on the left side increased with weight, a relationship that was not observed on the right side. Overall, age exerts the most widespread effect on these anatomical characteristics, followed by weight. The influence of BMI on these anatomical features primarily aligns with the effects of weight.

### 3.3 Impact of Anatomical Changes on Surgical Complications

In the univariate regression analysis for the CAS‐treated group, postoperative complications were significantly associated with symptomatic stenosis, variations in the aortic arch, CCA ostial lesions, aortic arch calcification level, tandem lesions, and CCA diameter (Table S2). For the CEA‐treated group, only symptomatic stenosis showed a significant association with postoperative complications. In the multivariate regression analysis for the CAS‐treated group, symptomatic stenosis, variations in the aortic arch, CCA ostial lesions, and CCA diameter remained significantly associated with postoperative complications. Based on this multivariate analysis, along with stepwise multivariate regression, a predictive model for the complication rate in the CAS‐treated group was developed:

$$\begin{array}{c} \text{Complication Rate}=0.462+0.386^{*}O—0.074^{*}D+0.161^{*}S\\ \;+0.431^{*}V \end{array}$$

where *O* represented CCA ostial lesions, *D* represented CCA diameter, *S* represented symptomatic stenosis, and *V* represented variations in the aortic arch.

**FIGURE 3:**
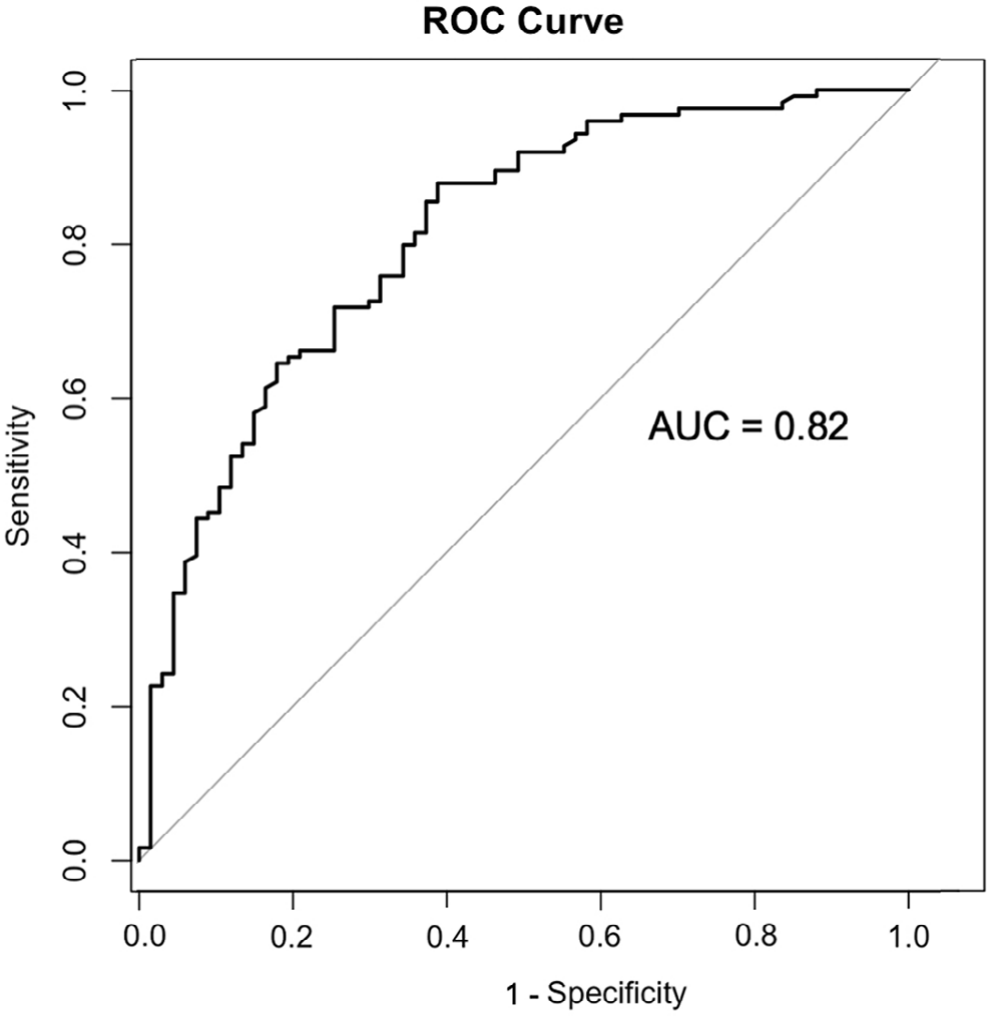
ROC curve of the logistic prediction model for complications after carotid artery stenting. The model demonstrates a discriminatory power with an AUC of 0.82.

The model demonstrated good discriminatory power with an AUC of 0.82 (Figure 3). Visualization of the prediction model was achieved through a nomogram (Figure S1). The calibration curve showed that the predicted complication rates closely matched the observed rates, with minimal deviation from the 45° line, indicating good calibration (Figure S2). Decision curve analysis further demonstrated the model's clinical utility, with positive net benefits across a range of threshold probabilities. The net benefit curves consistently outperformed the “None” and “All” strategies, confirming favorable clinical applicability (Figure S3).

In comparison, various machine learning models were evaluated. The decision tree model achieved an AUC of 0.66, the SVM model reached an AUC of 0.69, the RF model had an AUC of 0.63, and the GBM model yielded an AUC of 0.80 (Figure S4). Among these, the logistic regression model and the GBM model exhibited the highest performance, with the logistic regression model showing slightly better discrimination.

## 4 Discussion

In our study, we observed that age plays a significant role in shaping the anatomical characteristics of the carotid arteries. Specifically, the diameter of the CCA increases with age, while the distance between the clavicle and carotid bifurcation decreases. Additionally, both the CCA and ICA demonstrate an increase in tortuosity as age progresses. These findings align with previous research that highlights several age‐related changes in vascular anatomy, such as the enlargement of vessel volumes, increased diameters of the CCA and ICA, and changes in the bifurcation angle and rotation of the carotid artery. Moreover, age is associated with a decline in hemodynamic parameters, including wall shear stress, velocity, pressure gradient, and energy loss [21, 22]. Compared to reports worldwide, Chinese populations tend to exhibit more complex aortic arch morphologies, including a higher frequency of Type III arches (28.9%), greater calcification of the aortic arch and its branches [32, 38], and an increased tortuosity index for both the CCA and ICA [31]. Those factors may complicate stent navigation and placement during CAS procedures. These anatomical alterations may contribute to the increasing surgical risks observed in older patients, as changes in vascular geometry can lead to complications during procedures. The progressive widening and tortuosity of the carotid artery, along with the changes in the vessel diameter, are particularly important considerations when assessing surgical outcomes. Consequently, understanding these age‐related anatomical features is essential for better predicting postoperative risks and optimizing treatment strategies in elderly patients.

Our findings emphasize the role of age‐related anatomical changes in the risk of postoperative complications. The increased CCA diameter, presence of CCA ostial lesions, and variations in the aortic arch, all of which have been identified as key anatomical predictors, are strongly associated with complication risks. These results are consistent with previous studies that have demonstrated the significant impact of such features on procedural outcomes in carotid stenting [39, 40, 41]. Notably, the significant association between these anatomical characteristics and postoperative complications in the CAS‐treated group, but not in the CEA‐treated group, highlights the distinct challenges posed by CAS in patients with these anatomical features.

The disparity between CAS and CEA outcomes further underscores the differential risks posed by each technique. In contrast to CAS, symptomatic stenosis emerged as the sole significant predictor of complications in the CEA group. This distinction may be explained by the inherent differences in procedural mechanisms. CAS is more technically demanding and highly dependent on vascular anatomy, particularly in elderly patients who commonly exhibit complex vessel morphology. Tortuous or heavily calcified vessels can compromise catheter navigation and stent deployment, increasing the risk of embolization or inadequate lesion coverage. In contrast, CEA is a surgical procedure that allows direct visualization and removal of the plaque, thereby reducing the impact of anatomical variability. Additionally, differences in perioperative hemodynamic stress may be a potential contributor [42]. CAS often triggers more pronounced baroreflex activation and fluctuations in cerebral perfusion, which may further exacerbate complications in anatomically challenging cases.

This indicates that CEA may be better suited for elderly patients due to its more direct approach in plaque removal, reducing distal embolization risk. The increased vascular stiffness in elderly patients makes the passage of the CAS guidewire more difficult, elevating the risk of embolic events. Furthermore, the need for anticoagulation and antiplatelet therapy in CAS presents an additional bleeding risk for this age group. On the other hand, CAS may be more advantageous for younger patients, who typically have better vascular elasticity. Younger individuals also tend to tolerate antiplatelet therapy better and experience higher long‐term stent patency. The higher prevalence of Type III arches, aortic arch calcification, and increased vascular tortuosity in Chinese patients may necessitate more meticulous procedural planning, particularly for CAS. These anatomical variations can complicate endovascular navigation and stent deployment. Thus, the choice between CEA and CAS should be guided by age and anatomical characteristics, with the understanding that age‐related changes may complicate CAS procedures, particularly in older populations. Our findings highlight the importance of anatomical features, such as CCA diameter, tortuosity, and bifurcation level, in predicting procedural risks, particularly for CAS. Translating these insights into clinical practice supports the consideration of more advanced imaging modalities as part of routine preoperative assessment. Advanced techniques such as 4D flow MRI have shown the ability to noninvasively assess both anatomical structure and hemodynamic parameters [21]. This approach may enhance individualized risk stratification and guide treatment selection in patients with anatomically challenging carotid lesions. Although anatomical differences between Chinese and Western populations have been noted, current Chinese guidelines largely follow Western recommendations without specific adaptations based on population‐specific vascular anatomy [43]. This represents an important gap and a potential area for future refinement of regionally tailored clinical protocols.

This study developed a multivariate predictive model for postoperative complications in the CAS‐treated group, demonstrating good discriminatory power (AUC = 0.82) and favorable calibration. The model highlighted the importance of key anatomical features, including CCA diameter, symptomatic stenosis, CCA ostial lesions, and variations in the aortic arch, as significant predictors of postoperative complications. This emphasizes the need for careful preoperative assessment of these anatomical factors, particularly in patients undergoing CAS. While previous studies suggested that machine learning methods often outperform traditional logistic regression in predicting complications for carotid artery stenosis [41], our study found that the AUC of the logistic regression model consistently exceeded that of most machine learning models, except for the GBM model, which approached the AUC of the logistic regression model. The integration of age‐related anatomical changes into the predictive model further underscores the critical role these features play in determining surgical outcomes. By incorporating age‐related anatomical predictors, our predictive model advances the preoperative evaluation process, helping clinicians identify high‐risk patients and make more informed decisions regarding surgical interventions.

This study has certain limitations. The retrospective design may introduce selection bias, and the findings are specific to a Chinese population. Future studies should aim to validate our predictive model in larger, multi‐ethnic cohorts and explore the potential for incorporating additional variables.

## 5 Conclusion

Our study highlights the significant impact of age on the anatomical characteristics of patients with carotid artery stenosis, in terms of vascular tortuosity index, arterial diameter, and distance between the clavicle and carotid bifurcation. These unique anatomical characteristics are closely associated with postoperative complications, especially in patients undergoing CAS. Given the influence of age‐related anatomical changes, further research is needed to validate these findings in broader populations, particularly in older patients.

## Author Contributions

All authors of this manuscript meet the authorship criteria of the International Committee of Medical Journal Editors (ICMJE). Specifically, each author has made substantial contributions to: Conceptualization: Xiao Zhang, Liqun Jiao, Tao Wang; Writing – Original Draft: Xiao Zhang, Jia Zhou, Renjie Yang; Writing – Review and Editing: Xiao Zhang, Jia Zhou, Tao Wang; Methodology: Xiao Zhang, Jia Zhou, Renjie Yang, Wenjing Li; Project Administration: Xiao Zhang, Liqun Jiao, Tao Wang; Formal Analysis: Jia Zhou, Renjie Yang; Validation: Jia Zhou; Investigation: Renjie Yang, Xinyu Yang, Xiangyu Li; Data Curation: Jiaqi Jin, Yong Zeng, Yixin Sun, Zixuan Xing; Resources: Shuaiwei Guo, Jiayao Li, Shengyan Cui; Supervision: Wenjing Li, Xiaoli Min, Liqun Jiao, Tao Wang; Funding Acquisition: Xiaoli Min, Tao Wang.

## Ethics Statement

The study procedures were approved by the Ethics Committee of Xuanwu Hospital (ethics number: [2021]124) under the Declaration of Helsinki.

## Consent

The committee determined that as this study was retrospective and all included patients remain anonymous, patient informed consent is not required for this study.

## Conflicts of Interest

The authors declare no conflicts of interest.

## Supporting information


Appendix S1.
**Figure S1.** Nomogram of the logistic prediction model for complications after carotid artery stenting. The prediction model is visualized to facilitate the estimation of risk levels for post‐surgery complications based on key clinical variables.
**Figure S2.** Calibration plot of the logistic prediction model for complications after carotid artery stenting. Minimal deviation from the 45° line indicates good calibration.
**Figure S3.** Decision curve analysis of the logistic prediction model for complications after carotid artery stenting. The net benefit curves confirm the favorable clinical applicability of the model.
**Figure S4.** ROC curve of the prediction model under gradient boosting machines for complications after carotid artery stenting. The model demonstrates a discriminatory power with an AUC of 0.80.

**Table S1.** Baseline characteristics of study population.

**Table S2.** Variables significantly associated with postoperative complications under univariate regression. Table S1. Baseline characteristics of patients presenting with carotid artery stenosis.

## Data Availability

The data that support the findings of this study are available from the corresponding author upon reasonable request.
