# Supplementary material for: Age‐Related Anatomical Changes in Carotid Artery Stenosis and Its Impact on Postoperative Complications in Stenting and Endarterectomy
Source: CNS Neurosci Ther. 2025 Jul 23;31(7):e70527. doi: 10.1111/cns.70527 (PMC12287541; doi:10.1111/cns.70527)
Supplement: Supplementary file 1 — Appendix S1. Figure S1. Nomogram of the logistic prediction model for complications after carotid artery stenting. The prediction model is visualized to facilitate the estimation of risk levels for post‐surgery complications based on key clinical variables. Figure S2. Calibration plot of the logistic prediction model for complications after carotid artery stenting. Minimal deviation from the 45° line indicates good calibration. Figure S3. Decision curve analysis of the logistic prediction model for complications after carotid artery stenting. The net benefit curves confirm the favorable clinical applicability of the model. Figure S4. ROC curve of the prediction model under gradient boosting machines for complications after carotid artery stenting. The model demonstrates a discriminatory power with an AUC of 0.80. [file CNS-31-e70527-s002.docx]

**Supplementary material**

**Age-Related Anatomical Changes in Carotid Artery Stenosis and Its Impact on Postoperative Complications in Stenting and Endarterectomy**

Xiao Zhang^1,2 †^, PhD, Jia Zhou^3, †^, MD, Renjie Yang^1,2 †^, MD, Jiaqi Jin^1,2^, MD, Yong Zeng^4^, PhD, Shuaiwei Guo^1,2^, MD, Jiayao Li^1,2^, MD, Yixin Sun^5,6^, MD, Zixuan Xing^7^, MD, Shengyan Cui^1,2^, MD, Xinyu Yang^1,2^, MD, Xiangyu Li^1,2^, MD, Wenjing Li^8,9^, PhD, Xiaoli Min^10^*, MD, PhD, Liqun Jiao^1,2,11^*, MD, Tao Wang^1,2^*, MD





**Figure S1.** Nomogram of the logistic prediction model for complications after carotid artery stenting. The prediction model is visualized to facilitate the estimation of risk levels for post-surgery complications based on key clinical variables.





**Figure S2.** Calibration plot of the logistic prediction model for complications after carotid artery stenting. Minimal deviation from the 45-degree line indicates good calibration.





**Figure S3.** Decision curve analysis of the logistic prediction model for complications after carotid artery stenting. The net benefit curves confirm the favorable clinical applicability of the model.





**Figure S4.** ROC curve of the prediction model under gradient boosting machines for complications after carotid artery stenting. The model demonstrates a discriminatory power with an AUC of 0.80.

**Table S1.** Baseline characteristics of study population

| **Baseline Characteristics** | | **Patients (N = 470)** |
| --- | --- | --- |
| **Demographic characteristics** | |  |
|  | Age, mean (±SD), years | 64.6±8.1 |
|  | Male, n (%) | 406 (86.4%) |
|  | Female, n (%) | 64 (13.6%) |
|  | Height, (±SD), cm | 167.8±8.0 |
|  | Weight, (±SD), kg | 71.4±11.5 |
|  | BMI, mean (±SD), kg/m^2^ | 25.8±15.0 |
| **Comorbidities** | |  |
|  | Other cardiovascular diseases (%) | 355 (75.5%) |
|  | Metabolic disorders (%) | 304 (64.7%) |
|  | Respiratory diseases (%) | 3 (0.6%) |
|  | Gastrointestinal conditions (%) | 5 (1.1%) |
|  | Renal/hepatic dysfunction (%) | 3 (0.6%) |
|  | Hematologic disorders (%) | 1 (0.2%) |
| **Smoking history** | |  |
|  | Never smoked (%) | 201 (42.8%) |
|  | Currently smoking (%) | 148 (31.5%) |
|  | Former smoker (%) | 121 (25.7%) |
| **Alcohol use history** | |  |
|  | Never drank alcohol (%) | 261 (55.5%) |
|  | Currently drinking (%) | 149 (31.7%) |
|  | Former drinker (%) | 60 (12.8%) |

**Abbreviation:** BMI, body mass index; SD, standard deviation.

**Table S2.** Variables significantly associated with postoperative complications under univariate regression

| **Variables** | **Beta** | **S. E** | **Z** | **P** | **OR (95%CI)** |
| --- | --- | --- | --- | --- | --- |
| *CAS-treated group* | | | | | |
| **Symptomatic stenosis** |  |  |  |  |  |
| No |  |  |  |  | 1.000 (Reference) |
| Yes | 0.905 | 0.329 | 2.749 | 0.006 | 2.471 (1.313-4.796) |
| **Variations in the aortic arch** |  |  |  |  |  |
| No |  |  |  |  | 1.000 (Reference) |
| Yes | 2.091 | 0.536 | 3.901 | ＜0.001 | 8.092 (3.017-25.712) |
| **CCA ostial lesions** |  |  |  |  |  |
| No |  |  |  |  | 1.000 (Reference) |
| Yes | 1.752 | 0.385 | 4.553 | ＜0.001 | 8.092 (2.760-12.582) |
| **Aortic arch calcification level** |  |  |  |  |  |
| I-III |  |  |  |  | 1.000 (Reference) |
| IV | 1.170 | 0.350 | 3.338 | ＜0.001 | 3.221 (1.655-6.585) |
| **Tandem lesions** |  |  |  |  |  |
| No |  |  |  |  | 1.000 (Reference) |
| Yes | 0.839 | 0.329 | 2.551 | 0.011 | 2.314 (1.215-4.429) |
| **CCA diameter** | -0.508 | 0.157 | -3.227 | 0.001 | 0.602 (0.437-0.811) |
| *CEA-treated group* | | | | | |
| **Symptomatic stenosis** |  |  |  |  |  |
| No |  |  |  |  | 1.000 (Reference) |
| Yes | 0.8666 | 0.3423 | 2.532 | 0.011 | 2.379 (1.242-4.790) |

**Abbreviation:** CCA, common carotid artery; CEA, carotid endarterectomy; CI, confidence interval; ICA, internal carotid artery; OR, odds ratio; S. E, standard error; CAS, carotid artery stentin.
